# Analgesic Efficacy and Safety of Intrathecal Morphine or Intercostal Levobupivacaine in Lung Cancer Patients after Major Lung Resection Surgery by Videothoracoscopy: A Prospective Randomized Controlled Trial

**DOI:** 10.3390/jcm13071972

**Published:** 2024-03-28

**Authors:** Silvia González-Santos, Borja Mugabure, Manuel Granell, Borja Aguinagalde, Iker J. López, Ainhoa Aginaga, Inmaculada Zubelzu, Haritz Iraeta, Jon Zabaleta, Jose Miguel Izquierdo, Nuria González-Jorrín, Cristina Sarasqueta, Alejandro Herreros-Pomares

**Affiliations:** 1 Department of Anesthesiology, Postoperative Care and Pain Management, Donostia University Hospital, 20014 San Sebastián, Spain; dra_sgsantos@yahoo.es (S.G.-S.); mugabure@yahoo.es (B.M.); mariaainhoa.aginagabadiola@osakidetza.eus (A.A.); mariainmaculada.zubelzujaca@osakidetza.eus (I.Z.); haritz.iraetaoregi@osakidetza.eus (H.I.); nuria.gonzalezjorrin@osakidetza.eus (N.G.-J.); 2 Department of Anesthesiology, Postoperative Care and Pain Management, Hospital General Universitario de València, 46014 Valencia, Spain; 3 Department of Surgery, Universitat de València, 46010 Valencia, Spain; 4 Department of Thoracic Surgery, Donostia University Hospital, 20014 San Sebastián, Spain; borja.aguinagaldevaliente@osakidetza.eus (B.A.); ikerjavier.lopezsanz@osakidetza.eus (I.J.L.); jon.zabaletajimenez@osakidetza.eus (J.Z.); josemiguel.izquierdoelena@osakidetza.eus (J.M.I.); 5 Department of Clinical Epidemiology, Donostia University Hospital, ISS Bioguipuzcoa, 20014 San Sebastián, Spain; cristina.sarasquetaeizaguirre@osakidetza.eus; 6 Department of Biotechnology, Universitat Politècnica de València, 46022 Valencia, Spain; alherpo@etsiamn.upv.es; 7 Centro de Investigación Biomédica en Red Cáncer, CIBERONC, 28029 Madrid, Spain

**Keywords:** video-assisted thoracoscopic surgery, intrathecal morphine, intercostal levobupivacaine

## Abstract

**Background**: Lung resection using video-assisted thoracoscopic surgery (VATS) improves surgical accuracy and postoperative recovery. Unfortunately, moderate-to-severe acute postoperative pain is still inherent to the procedure, and a technique of choice has not been established for the appropriate control of pain. In this study, we aimed to compare the efficacy and safety of intrathecal morphine (ITM) with that of intercostal levobupivacaine (ICL). **Methods**: We conducted a single-center, prospective, randomized, observer-blinded, controlled trial among 181 adult patients undergoing VATS (ISRCTN12771155). Participants were randomized to receive ITM or ICL. Primary outcomes were the intensity of pain, assessed by a numeric rating scale (NRS) over the first 48 h after surgery, and the amount of intravenous morphine used. Secondary outcomes included the incidence of adverse effects, length of hospital stay, mortality, and chronic post-surgical pain at 6 and 12 months after surgery. **Results**: There are no statistically significant differences between ITM and ICL groups in pain intensity and evolution at rest. In cough-related pain, differences in pain trajectories over time are observed. Upon admission to the PACU, cough-related pain was higher in the ITM group, but the trend reversed after 6 h. There are no significant differences in adverse effects. The rate of chronic pain was low and did not differ significantly between groups. **Conclusions**: ITM can be considered an adequate and satisfactory regional technique for the control of acute postoperative pain in VATS, compatible with the multimodal rehabilitation and early discharge protocols used in these types of surgeries.

## 1. Introduction

Video-assisted thoracoscopic surgery (VATS) is a minimally invasive procedure currently employed in major thoracic surgical procedures following enhanced recovery after surgery (ERAS) protocols. The transition from conventional anesthesia practices to less invasive approaches eases faster postoperative recoveries, but it needs a multidisciplinary approach, with anesthetists playing a pivotal role [1,2,3]. Unfortunately, moderate-to-severe acute postoperative pain remains inherent to the procedure, and inadequate postoperative pain control is associated with poorer postoperative recovery in terms of lung complications and a non-negligible risk of developing chronic pain [4,5,6].

Thoracic epidural analgesia (TEA) and paravertebral block (PVB) currently stand as the gold standard for managing postoperative pain after major thoracic surgery via thoracotomy. Additionally, PVB has been adopted as the standard analgesia after VATS [7,8] due to its ability to provide optimal analgesia with fewer side effects than TEA [9]. While TEA achieves excellent postoperative analgesia, it is incompatible with the enhanced recovery and early discharge programs offered by VATS, primarily due to side effects such as urinary retention. Various regional techniques have been proposed for pain management during and after VATS, but a definitive technique of choice has not yet been established [9,10,11,12,13,14]. For example, studies have demonstrated the non-inferiority of intercostal block compared to TEA in terms of analgesic quality or preservation of postoperative lung function after VATS [15]. Moreover, both PVB and intercostal nerve blocks have been successfully used for pain management in this type of surgery [16,17,18].

Intrathecal morphine (ITM) block, also known as spinal analgesia, is another less commonly used analgesic method during lung surgery. This involves administering a morphine injection into the spinal subarachnoidal space to provide pain relief [19,20,21]. Since 2014, we have successfully employed ITM in patients scheduled for VATS in our hospital. To the best of our knowledge, there are no methodologically relevant studies in the scientific literature comparing it with locoregional blocks and patient-controlled analgesia with intravenous (IV) opioids in thoracoabdominal surgery, despite ITM administration being an easily learned technique that is both safe and cost-effective. Due to its advantages over new locoregional techniques, which require additional training and ultrasound equipment [20,21,22,23], ITM has been proposed as an alternative for VATS patients included in ERAS programs by some authors [24].

The scarcity of studies on this topic prompted us to conduct a clinical trial comparing ITM with an intercostal nerve block (ICNB) with local anesthetics, which is one of the regional block techniques that appears more frequently in the clinical literature in VATS and is recommended by the guidelines for Enhanced Recovery after Lung Surgery: recommendations of the ERAS Society and the European Society of Thoracic Surgeons (ESTS) [1]. This study aims to compare the effectiveness and safety of these two techniques after lung resection in VATS.

## 2. Materials and Methods

This single-center, observer-blinded, randomized trial received approval from the Institutional Review Board (Ethics Committee of the Basque Country) on 6 November 2017 and from the Spanish Agency of Medicines and Medical Devices on 13 November 2017. Written informed consent was obtained from all subjects before the commencement of this study. The clinical trial was pre-registered at the ISRCTN Registry (ISRCTN12771155, https://doi.org/10.1186/ISRCTN12771155; Principal Investigator: Dr. Silvia González-Santos; Date of registration: 30 December 2016).

The inclusion criteria comprised patients aged 18 years or older with an ASA physical status from 1 to 4 (males and females), scheduled to undergo major lung resection (bilobectomy, lobectomy, or segmentectomy) via VATS. Exclusion criteria included the absence of written consent, a history of drug abuse, chronic pain treated with opioids, or any contraindications to spinal or intercostal nerve block (bleeding disorders, recent systemic or local infections, allergy to local anesthetics or morphine, expansive processes of the central nervous system, hydrocephalus, or lumbar spinal conditions contraindicating lumbar puncture), as well as unexpected conversion from minimally invasive thoracic surgery to open thoracotomy.

A total of 181 eligible patients were enrolled and randomly allocated in a 1:1 ratio to receive intercostal levobupivacaine (ICL) or ITM using a computer-generated random allocation sequence created by the study statistician. Allocation numbers were sealed in opaque envelopes, which were opened sequentially by the anesthesiologist in charge of the patient. All the researchers involved in the postoperative evaluation and treatment of the patients were different from those in the operating room and were unaware of the analgesic technique used during the surgery. Additionally, a dressing was placed on the back of all patients to ensure that those who had received spinal block with morphine could not be identified based on the presence of such a dressing.

### 2.1. Trial Design

All patients underwent standardized premedication, monitoring, and administration of general anesthesia. General anesthesia induction included intravenous fentanyl at a dose of 2 µg/kg, followed by propofol at 2–3 mg/kg and rocuronium at 0.6 mg/kg. Tracheal intubation was performed using a left-sided double-lumen endotracheal tube whenever possible; alternatively, a single-lumen endotracheal tube with a built-in blocker (UniventTM) was employed. Ventilation settings were adjusted to a tidal volume of 6–8 mL/kg of ideal body weight during the two-lung ventilation period. Anesthesia maintenance involved sevoflurane (targeting a bispectral index between 40 and 60), intravenous fentanyl based on changes in vital signs (administering 1 µg/kg if systolic blood pressure > 140 mmHg and/or heart rate > 100 beats/min), and continuous intravenous infusion of rocuronium (0.4 mg/kg/h). Throughout the surgery, patient analgesia was supplemented with intravenous paracetamol 1 g and dexketoprofen 50 mg. Additionally, 4 mg intravenous ondansetron was administered as an antiemetic. Urinary catheterization was utilized for diuresis control after anesthetic induction and removed within 24 h before discharge from the postanesthesia care unit (PACU).

In the ICL group, ICNB was performed at the end of surgery, right before skin closure, using levobupivacaine 0.5% at a dose of 2 mg/kg (up to a maximum of 150 mg). The block was administered at the level of the main chest incision, two levels above and two levels below it, and at the level of the chest where the drain was inserted at the end of the intervention. Patients in the ITM group received an intrathecal injection of morphine hydrochloride before anesthesia induction using a 25-gauge pencil-tip needle at the L2–L3 or L3–L4 level. The dose was height-adjusted (150 µg for height < 1.60 m; 200 µg for height 1.60–1.80 m; and 250 µg for height > 1.80 m).

All surgeries in both groups were performed with patients in the lateral decubitus position using a 10 mm trocar and an accessory incision. The trocar was positioned in the 7th–8th intercostal space, and the accessory incision (4 cm) was made in the 4th–5th intercostal space in the anterior axillary line. No rib spreader was used. Closure was performed in the muscular and subcutaneous plane without closing the intercostal space. A 24F silicone thoracic drain (Redax^®^, Poggio Rusco, Italy) was placed in the trocar orifice, and drainage removal occurred when no leakage was observed in water seal chambers or digital systems.

Following surgery, patients were transferred to the PACU for monitoring. We recorded various variables upon PACU arrival (0 h) and at 6, 12, 24, and 48 h post-surgery. These included pain at rest and on coughing assessed by a numeric rating scale (NRS), incidence of nausea, vomiting, pruritus, and respiratory depression, level of sedation according to the Richmond Agitation–Sedation Scale (RASS), and any cardiac or respiratory complications. As per the protocol, patients received intravenous paracetamol (1 g/8 h), dexketoprofen (50 mg/12 h), and metamizole (2 g/8 h) for 48 h. Boluses of 0.05 mg/kg intravenous morphine were administered on demand if the NRS score was over 3. Stomach protection medication (40 mg intravenous omeprazole/24 h) and antiemetic medication (4 mg/8 h intravenous ondansetron and 0.0625 mg intravenous droperidol as rescue antiemetic therapy) were also prescribed.

### 2.2. End Points and Assessments

The primary outcome measures included postoperative pain, assessed by patients using the NRS on a scale ranging from 0 to 10, at 0, 6, 12, 24, and 48 h after surgery both at rest and on coughing, and the total amount of rescue intravenous (IV) morphine used during the first 24 h.

The secondary outcome measures comprised the following: incidence of postoperative nausea and/or vomiting (PONV); respiratory depression, identified by pulse oximetry (peripheral oxygen saturation/SpO_2_ < 90%) and continuous monitoring of respiratory rate (<10 breaths/min); level of sedation, evaluated using the Richmond Agitation–Sedation Scale (RASS); occurrence of pruritus; respiratory complications (atelectasis requiring fiberoptic bronchoscopy, pneumonia, respiratory failure necessitating endotracheal intubation); cardiac complications (atrial fibrillation, acute myocardial infarction, congestive heart failure); urinary retention requiring re-catheterization or difficulty removing the urinary catheter after 24 postoperative hours; 90-day mortality; and length of hospital stay.

Furthermore, subjects were contacted by telephone to inquire about any persistent pain or discomfort at specific sites (VATS ports, anterior or posterior thorax, axilla, or ipsilateral upper limb) 6 months post-surgery. In cases where patients reported chronic post-surgical pain, the nature and intensity of the pain were assessed using the Brief Pain Inventory (BPI) questionnaire at that time and again at 12 months after the surgery.

### 2.3. Statistical Analysis

Graphical analysis, assessment of kurtosis and skewness, and the Kolmogorov–Smirnov test were used to assess the distribution of the data. 

For the descriptive analysis, means and standard deviations were calculated if the data were normally distributed; otherwise, medians and interquartile ranges (IQR) were used. Chi-square tests were applied for percentages, and Student’s *t*-test and Mann–Whitney U test were used for continuous variables that were and were not normally distributed, respectively.

For both pain at rest and pain on coughing, a two-way repeated measures ANOVA was utilized to compare the mean differences between groups (type of treatment) and the interaction between group and time (pain at five time points). This analysis assessed whether there were differences between groups in the evolution of pain throughout the 48 h of follow-up. Bonferroni correction was applied for post hoc comparisons. To analyze differences between groups in pain at 6 and 12 months, the Mann–Whitney U test was employed.

A sensitivity analysis was conducted to explore the impact of missing data on the results. The results were compared under the assumption of the best-case scenario (absence of pain in missing data) and the worst-case scenario (maximum pain in missing data).

To determine an appropriate sample size, an a priori calculation was performed using mean pain scores on the NRS of 2.5 and 3.5 in the ITM and ICL groups, respectively. A sample size of 174 patients was deemed sufficiently large with a statistical power of 80%, considering a two-tailed *t*-test and an alpha of 0.05 (Power = 0.8, α = 0.05). We enrolled 244 patients to account for the potential loss of cases due to withdrawal, conversions to thoracotomy or minor resections, impossibility of applying the planned regional techniques, necessity of a second intervention, or other reasons.

Statistical analyses were performed using the Statistical Package for the Social Sciences (SPSS, Chicago, IL, USA) version 29.0.

## 3. Results

**Figure 1:**
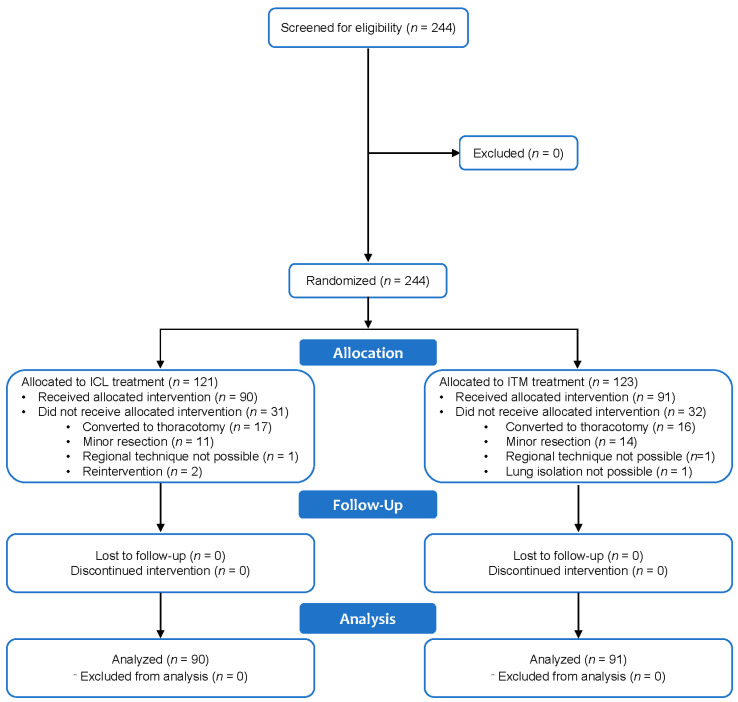
Consolidated Standards of Reporting Trials (CONSORT) flowchart. ICL, intercostal levobupivacaine; ITM, intrathecal morphine.

Figure 1 illustrates the Consolidated Standards of Reporting Trials (CONSORT) flow diagram. Between December 2017 and June 2020, patients who met the inclusion criteria were randomly assigned to receive ITM or ICL in a 1:1 allocation ratio. A total of 244 patients were screened for eligibility, and 181 met all trial requirements. Sixty-three patients did not receive allocated intervention for various reasons, including conversion to thoracotomy during surgery (*n* = 33), conversion to minor resection (*n* = 25), requirement of a second intervention (*n* = 2), failure to perform lumbar puncture (ITM group, *n* = 1), inability to perform ICL due to tumor invasion of the costal wall (ICL group, *n* = 1), or impossibility of achieving lung isolation (*n* = 1) (Figure 1). There were no losses to follow up in either of the study groups.

The clinical profile and surgical information of the 181 included patients are presented in Table 1. The mean patient age was 64 years; more than 66% were males, and over 98% were classified as ASA II-III. The mean intraoperative (IO) fentanyl used was similar for both groups, and no clinically relevant differences were detected between the groups. The average surgical time was 3.2 h, with upper right lobectomy (RUL) being the most common type of resection and middle lobectomy (ML) the least common.

### 3.1. Primary Outcome Data

The pain averages in the first 48 postoperative hours are presented in Table 2. Pain experienced by patients at rest and on coughing was assessed by a numeric rating scale (NRS—on which patients rated their level of pain between 0 and 10) at 0, 6, 12, 24, and 48 h after surgery for the control and the interventional groups.

Upon admission to the PACU (0 h), pain was higher in the ITM group than in the ICL group at rest [3.6 (2.9) vs. 2.4 (2.9), *p* = 0.03] and on coughing [4.9 (2.9) vs. 3.4 (3.0), *p* = 0.01]. However, no differences in pain at rest were observed for the rest of the time points evaluated. On coughing, patients allocated to the ITM group experienced lower postoperative pain levels at 12 [3.5 (2.4) vs. 4.7 (2.3), *p* = 0.01) and 24 h [4.5 (2.2) vs. 5.5 (2.0), *p* = 0.01) than patients allocated to the ICL group.

**Figure 2:**
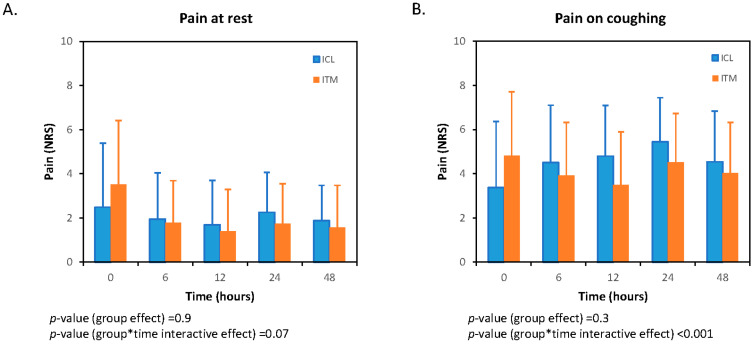
Evolution of pain means at 0, 6, 12, 24, and 48 h in both groups, at rest (**A**), and on coughing (**B**). ITM, intrathecal morphine; ICL, intercostal levobupivacaine. The asterisk indicates multiplication.

Repeated measures of ANOVA for pain postoperative at rest and on coughing are shown in Figure 2A and Figure 2B, respectively. There are no statistically significant differences between groups in pain intensity at rest; *p*(group) = 0.9. The differences in the evolution of pain at rest in the first 48 h do not reach statistically significant levels either; *p*(group × ime interaction) = 0.07 (Figure 2A). In cough-related pain, the group effect is not statistically significant; *p*(group) = 0.27. However, differences in pain trajectories over time are observed; *p*(group × time interaction) < 0.001 (Figure 2B).

**Figure 3:**
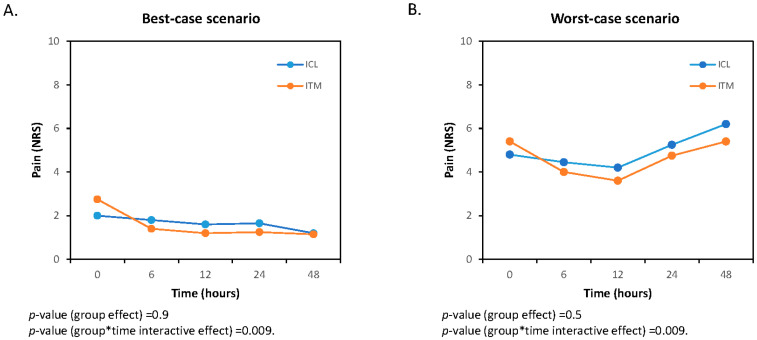
Best-case (**A**) and worst-case (**B**) scenarios of the sensitivity analysis for pain at rest. ITM, intrathecal morphine; ICL, intercostal levobupivacaine. The asterisk indicates multiplication.

**Figure 4:**
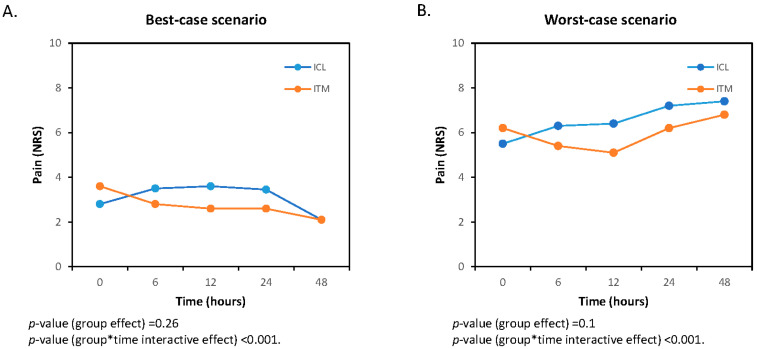
Best-case (**A**) and worst-case (**B**) scenarios of the sensitivity analysis for pain on coughing. ITM, intrathecal morphine; ICL, intercostal levobupivacaine. The asterisk indicates multiplication.

In the sensitivity analysis, statistically significant differences are observed in the interaction between time and treatment in both the evolution of pain at rest (Figure 3) and cough-related pain (Figure 4). However, there are no differences between groups in either scenario.

### 3.2. Intravenous Morphine Rescue

Analysis of the total amount of rescue IV morphine (coprimary endpoint) used during the first 24 h revealed no significant differences between the ITM and the ICL groups [6.9 (7.0) vs. 5.5 (5.9), *p* = 0.1] (Table 2).

### 3.3. Secondary Outcome Data

Safety summaries for ICL and ITM groups are presented in Table 3. The frequency of adverse events, including respiratory depression, PONV, pruritus, atelectasis, pneumonia, cardiac complications, urinary retention, and 90-day mortality, was similar in both groups. Moreover, there were no significant differences in the RASS score (4.1 for both groups) or the length of hospital stay (4.1 days for the ICL group vs. 3.9 days for the ITM group).

Eight patients in each group reported postoperative chronic pain after 6 months (risk ratio (RR) = 1, 95% confidence interval (CI) [0.39–2.55], *p* > 0.99). Notably, the incidence fell to six (6.7%) for the ICL group and three (3.3%) for the ITM group after 12 months (RR = 0.52, 95% CI [0.13–2.00], *p* = 0.34). The median intensity of the postoperative chronic pain in these patients was 4.0 at rest and 4.5 on coughing for the ICL group and 5.5 at rest and 6.5 on coughing for the ITM group after 6 months (Table 4). Furthermore, pain scores at rest and on coughing were, respectively, 4 and 4.5 in the ICL group and 4.5 and 7.0 in the ITM group after 12 months. No significant differences were found between the postoperative chronic pain in the ITM and the ICL groups.

## 4. Discussion

Analgesia in thoracic surgery remains a significant challenge despite the progressive minimization of invasive procedures. Fast-track surgery programs and enhanced recovery after surgery (ERAS) protocols have been progressively implemented due to their demonstrated benefits in postoperative outcomes after VATS. However, the successful application of these programs requires effective analgesia, early patient mobilization, and prompt removal of drainage tubes and urinary catheters, among other factors [3,24,25,26].

The efficacy of ITM has been established for various surgical procedures, emerging as the neuraxial technique of choice for pain management after major surgery within multimodal analgesia in ERAS protocols [21,27,28]. It is recommended by esteemed medical societies, including the European Society of Regional Anaesthesia and Pain Therapy (ESRA), the National Institute for Health and Care Excellence, and the Society of Obstetric Anesthesiology and Perinatology [23]. Despite its benefits, there is a paucity of studies conducted specifically in the context of VATS.

In our study, we randomized 181 patients scheduled for VATS to receive either ITM or ICL. Our results indicate no significant differences in the management of pain intensity between treatments, but pain evolution differed between the two groups. Upon arrival at PACU, patients in the ITM group experienced higher pain levels, but approximately 6 h after PACU admission, this trend reversed. While these differences are observed both at rest and on coughing, the trend becomes more pronounced when assessing pain on coughing. This behavior aligns with the pharmacokinetic properties of morphine administered in the intrathecal space. Hydrophilic opioids, like morphine, bind more strongly with specific receptors of the dorsal horn in the spinal cord than lipophilic opioids. Specifically, morphine exhibits greater spinal bioavailability and intrathecal residence time, resulting in the peak analgesic effect at 6 h and lasting for 24 h. In the sensitivity analysis, the absence of a significant group effect is maintained, but a differential pattern is still observed in the evolution of pain during the 48-h follow-up period. Probably, an intercostal continuous infusion of local anesthetics or liposomal bupivacaine would be a better technique than a single-shot plane levobupivacaine intercostal block [17,18]. 

We also observed no significant differences in the total amount of IV rescue morphine used. The total amount of rescue IV morphine utilized in the ITM group was comparable to that reported by Vijitpavan et al. after the application of ITM in VATS [29]. However, contrary to our findings, these authors reported poorer pain management in the control group, which did not involve a locoregional anesthetic technique. The divergence in pain management for the control group between our study and that of Vijitpavan et al. may imply that the utilization of intercostal block after VATS is a more suitable anesthetic technique than the absence of locoregional blockade, as previously suggested [29].

In addition to efficacy studies, studies providing clinical safety regarding the use of low doses of ITM in VATS are necessary. This is crucial because profound and prolonged respiratory depressions have been reported after high doses of ITM, making postoperative vigilance at PACU for 24 h advisable [30,31]. Clinical practices could potentially change if positive results from studies, such as that of Vijitpavan et al., are confirmed, where they reported no respiratory depressions after the use of 200 μg of ITM in VATS [29]. In our study, excessive sedation was identified in two patients who did not require artificial ventilation or intubation immediately after admission to the PACU. This sedation was attributable in both cases to the residual effect of the anesthetic drugs and analgesics administered intraoperatively. Moreover, no delayed respiratory depression was detected, supporting the safety of low ITM and low ICL doses. Similarly, Rathmell et al. and Cohen et al. reported that analgesic efficacy was achieved with up to 300 µg of ITM and estimated that the therapeutic ceiling effect would be reached with this maximum dose, beyond which the risk of adverse effects could outweigh the benefits of the analgesia provided [20,30]. Future clinical trials assessing the safety of low doses of ITM are still encouraged, with a focus on the incidence of delayed respiratory depression. Recently, Rawal suggested that patients receiving low-dose ITM could be cared for in regular surgical wards [23]. Monitoring recommendations from societies such as the ESRA, the American Society of Regional Anesthesia and Pain Medicine, and the American Society of Anesthesiologists need to be updated so that the requirements for extended or continuous monitoring in PACUs, step-down units, high-dependency units, and intensive care units can be eliminated for patients who underwent low doses of ITM. This would reduce additional costs and inconvenience, making this simple, versatile, and highly effective analgesia technique available to a wider patient population in resource-limited settings [23].

Regarding other adverse effects, there were no significant differences between groups. In the case of PONV, the incidence was slightly higher in the ITM group (not statistically significant), but the results obtained do not differ from those in other series [32]. However, preventive measures could be strengthened by increasing the use of antiemetics intraoperatively, for example, by administering IV high-dose corticoids, droperidol, etc., when intrathecal opioids are administered [33].

Regarding postoperative chronic pain, 16 patients, 8 patients treated with ICL (8.9%), and 8 patients treated with ITM (8.8%) reported postoperative chronic pain after 6 months. Importantly, this incidence fell to six for the ICL group (6.7%) and three (3.3%) for the ITM after 12 months. These results align with those reported by Wildgaard et al. [4], who noted an incidence of postoperative chronic pain in 11% of patients using thoracic epidural analgesia or paravertebral block in VATS. In contrast, Bendixen et al. [34], Kampe et al. [35], and Bayman et al. [36] reported postoperative chronic pain rates of 38%, 39%, and 47%, respectively. We hypothesize that the improvement in the management of postoperative chronic pain could be related not only to the analgesic methods but also to the characteristics of the surgical technique used, including no use of a rib spreader and no closure of the intercostal space. These factors have been previously described as contributing factors to achieving lower incidence rates of chronic pain [37]. Even if the difference between both locoregional analgesic techniques was not statistically significant at 12 months, which can be attributable to the sample size calculated to compare pain as rated by patients on the NRS, these findings could have clinical relevance if further studies confirm that the usage of ITM reduces the incidence of postoperative chronic pain compared to ICL or other analgesic strategies. In that sense, Bayman et al. [36] suggested that effective control of acute postoperative pain could considerably reduce postoperative chronic pain.

Limitations. This is a single-center study that reflects the experience of a single group of professionals at one hospital, so caution should be exercised in generalizing the findings. The sample size was calculated to compare pain as rated by patients on the NRS between treatments based on our previous experience. Unfortunately, a pilot study was not run to estimate the mean pain scores, which would have been a more appropriate approach. Further studies should be conducted to thoroughly investigate other outcomes, such as the incidence of postoperative chronic pain. The low number of patients reporting postoperative chronic pain compromises the reliability of the negative findings in this regard. The delayed publication of this paper is attributed to the COVID-19 pandemic. Finally, there are no studies about ITM in VATS, and it probably could be interesting to promote future trials comparing the ITM with other locoregional blocks such as TEA, paravertebral block, erector spinae plane block, or continuous intercostal block.

## 5. Conclusions

In summary, the results of this study support the use of low doses of ITM as a technique comparable to ICL in terms of analgesic efficacy, with a notable difference in the evolution of pain on coughing between the two groups. Initially, pain intensity was higher in the ITM group, but this trend reversed 6 h after PACU admission and disappeared in 48 h. Both locoregional analgesic methods used in our study effectively reduced acute pain and postoperative chronic pain. Although no respiratory depression was seen in the ITM group, further clinical trials are needed to determine whether patients undergoing ITM require surveillance in intermediate care for 24 h or if a hospital ward is a safe option for them. Combining both methods of locoregional analgesia could be an interesting protocol to explore, leveraging the rapid and brief analgesic effect of the ICL with the prolonged effect of ITM. Such a combined approach might facilitate adherence to fast-track programs for patients undergoing pulmonary resection using VATS. 

## Figures and Tables

**Table 1:** Clinical characteristics and surgery information of the patients included in this study.

Characteristics	ICL Group		ITM Group	
	*n* = 90	%	*n* = 91	%
Patient characteristics				
Age at surgery (years)	64.3 ± 9.1		64.9 ± 9.1	
Gender				
Male	60	66.7	60	65.9
Female	30	33.3	31	34.1
BMI (kg/m^2^)	25.8 ± 3.9		26.5 ± 4.1	
ASA physical status				
I	0	0.0	0	0.0
II	14	15.6	7	7.7
III	75	83.3	83	91.2
IV	1	1.1	1	1.0
Surgical information				
Surgical time (min)	192.2 ± 51.3		194.4 ± 57.5	
IO Fentanyl (µg)	497.2 ± 133.3		487.6 ± 131.1	
Duration of chest tube placement (days)	3.01 ± 3.2		2.71 ± 2.4	
Length of hospital stay (days)	4.1 ± 3.3		3.9 ± 3.2	
Type of surgery				
RUL	28	31.1	38	41.8
ML	3	3.3	7	7.7
RLL	15	16.7	14	15.4
LUL	16	17.8	9	9.9
LLL	12	13.3	8	8.8
TriSegmentectomy LSL	10	11.1	5	5.5
Segmentectomy S6 LIL	4	4.4	2	2.2
Segmentectomy S6 RIL	0	0.0	2	2.2
Segmentectomy S1 RSL	2	2.2	2	2.2
Lingulectomy	0	0.0	3	3.3
Segmentectomy S2 RSL	0	0.0	1	1.1

The central values indicate average ± SD. ASA, American Society of Anesthesiologists; BMI, body mass index; ICL, intercostal levobupivacaine; IO, intraoperative; ITM, intrathecal morphine; LLL, left lower lobectomy; LUL, left upper lobectomy; ML, middle lobectomy; RLL, right lower lobectomy; RUL, right upper lobectomy; LSI, left superior lobe; LII, left inferior lobe; RIL, right inferior lobe; RSL, right superior lobe.

**Table 2:** Assessment of pain intensity at 0, 6, 12, 24, and 48 h after surgery.

Postoperative Assessment	ICL (*n* = 90)	ITM (*n* = 91)	*p*-Value *
*Pain (NRS)*	Mean (SD)	Mean (SD)	
At rest			
0 h	2.4 (2.9)	3.6 (2.9)	0.03
6 h	1.9 (2.1)	1.8 (1.9)	0.85
12 h	1.7 (2.0)	1.4 (1.9)	0.48
24 h	2.3 (1.8)	1.8 (1.8)	0.12
48 h	1.9 (1.6)	1.6 (1.9)	0.31
On coughing			
0 h	3.4 (3.0)	4.9 (2.9)	0.01
6 h	4.4 (2.6)	3.9 (2.4)	0.27
12 h	4.7 (2.3)	3.5 (2.4)	0.01
24 h	5.5 (2.0)	4.5 (2.2)	0.01
48 h	4.6 (2.3)	4.0 (2.3)	0.21
*PO Morphine (mg)*			
	5.5 (5.9)	6.9 (7.0)	0.10

ICL, intercostal levobupivacaine; ITM, intrathecal morphine; NRS, numeric rating scale; PO, postoperative morphine; SD, standard deviation. * *p*-values related to pain are Bonferroni-corrected.

**Table 3:** Secondary outcomes, including treatment-related adverse events and chronic pain in all the patients treated with ICL or ITM.

Adverse Event	ICL *n* = 90 (%)	ITM *n* = 91 (%)	*p*-Value
Respiratory depression	1 (1.1)	1 (1.1)	1.00
PONV	16 (18.0)	26 (28.6)	0.10
Pruritus	1 (1.1)	1 (1.1)	1.00
Atelectasis	1 (1.1)	3 (3.3)	0.30
Pneumonia	0	3 (3.3)	0.10
Cardiac complications	0	0	-
Urinary retention	1 (1.1)	3 (3.3)	0.30
90-day mortality	1 (1.1)	1 (1.1)	1.00
Chronic pain (6 months)	8 (8.9)	8 (8.8)	0.80
Chronic pain (12 months)	6 (6.7)	3 (3.3)	0.30

ICL, intercostal levobupivacaine; ITM, intrathecal morphine; PONV, postoperative nausea and/or vomiting.

**Table 4:** Assessment of chronic pain intensity 6 and 12 months after surgery.

Chronic Pain Assessment	ICL Group (*n* = 90)	ITM Group (*n* = 91)
Number of patients reporting chronic pain	*n* (%)	*n* (%)
6 months	8 (8.9)	8 (8.8)
12 months	6 (6.7)	3 (3.3)
Pain (NRS) at rest	Median (IQR)	Median (IQR)
6 months	4.0 (3.3–4.8)	5.5 (3.0–8.0)
12 months	4.0 (3.3–4.8)	4.5 (3.0–8.0)
Pain (NRS) on coughing	Median (IQR)	Median (IQR)
6 months	4.5 (4.0–5.8)	6.5 (4.3–9.0)
12 months	4.5 (3.3–5.8)	7.0 (4.5–8.0)

ICL, intercostal levobupivacaine; 6 and 12 months after surgery; IQR, Interquartile range; ITM, intrathecal morphine; NRS, numeric rating scale.

## Data Availability

The data that support the findings of this study are available from the authors upon reasonable request.

## References

[1] Batchelor T.J.P., Rasburn N.J., Abdelnour-Berchtold E., Brunelli A., Cerfolio R.J., Gonzalez M., Ljungqvist O., Petersen R.H., Popescu W.M., Slinger P.D. (2019). Guidelines for enhanced recovery after lung surgery: Recommendations of the Enhanced Recovery after Surgery (ERAS^®^) Society and the European Society of Thoracic Surgeons (ESTS). Eur. J. Cardio-Thorac. Surg. Off. J. Eur. Assoc. Cardio-Thorac. Surg..

[2] Piccioni F., Ragazzi R. (2018). Anesthesia and analgesia: How does the role of anesthetists changes in the ERAS program for VATS lobectomy. J. Vis. Surg..

[3] Hung M.H., Chen J.S., Cheng Y.J. (2019). Precise anesthesia in thoracoscopic operations. Curr. Opin. Anaesthesiol..

[4] Wildgaard K., Ringsted T.K., Hansen H.J., Petersen R.H., Kehlet H. (2016). Persistent postsurgical pain after video-assisted thoracic surgery--an observational study. Acta Anaesthesiol. Scand..

[5] Zhang Y., Zhou R., Hou B., Tang S., Hao J., Gu X., Ma Z., Zhang J. (2022). Incidence and risk factors for chronic postsurgical pain following video-assisted thoracoscopic surgery: A retrospective study. BMC Surg..

[6] Wildgaard K., Ravn J., Nikolajsen L., Jakobsen E., Jensen T.S., Kehlet H. (2011). Consequences of persistent pain after lung cancer surgery: A nationwide questionnaire study. Acta Anaesthesiol. Scand..

[7] Yeung J.H.Y., Gates S., Naidu B.V., Wilson M.J.A., Gao Smith F. (2016). Paravertebral block versus thoracic epidural for patients undergoing thoracotomy. Cochrane Database Syst. Rev..

[8] Giang N.T., Van Nam N., Trung N.N., Anh L.V., Cuong N.M., Van Dinh N., Pho D.C., Geiger P., Kien N.T. (2018). Patient-controlled paravertebral analgesia for video-assisted thoracoscopic surgery lobectomy. Local Reg. Anesth..

[9] Raft J., Richebe P. (2019). Anesthesia for thoracic ambulatory surgery. Curr. Opin. Anaesthesiol..

[10] Moorthy A., Ní Eochagáin A., Dempsey E., Wall V., Marsh H., Murphy T., Fitzmaurice G.J., Naughton R.A., Buggy D.J. (2022). Postoperative recovery with continuous erector spinae plane block or video-assisted paravertebral block after minimally invasive thoracic surgery: A prospective, randomised controlled trial. Br. J. Anaesth..

[11] Campos J.H., Peacher D. (2020). Choosing the Best Method for Postoperative Regional Analgesia after Video-Assisted Thoracoscopic Surgery. J. Cardiothorac. Vasc. Anesth..

[12] Steinthorsdottir K.J., Wildgaard L., Hansen H.J., Petersen R.H., Wildgaard K. (2014). Regional analgesia for video-assisted thoracic surgery: A systematic review. Eur. J. Cardio-Thorac. Surg..

[13] Campos J.H., Seering M. (2019). Does the Amount of Opioid Consumption Really Matter in Video-Assisted Thoracoscopic Lobectomy-Thoracic Epidural Analgesia Versus Liposomal Bupivacaine. J. Cardiothorac. Vasc. Anesth..

[14] Finnerty D.T., McMahon A., McNamara J.R., Hartigan S.D., Griffin M., Buggy D.J. (2020). Comparing erector spinae plane block with serratus anterior plane block for minimally invasive thoracic surgery: A randomised clinical trial. Br. J. Anaesth..

[15] Ueda K., Hayashi M., Murakami J., Tanaka T., Utada K., Hamano K. (2020). Intercostal block vs. epidural analgesia in thoracoscopic lung cancer surgery: A randomized trial. Gen. Thorac. Cardiovasc. Surg..

[16] Zheng Y., Wang H., Ma X., Cheng Z., Cao W., Shao D. (2020). Comparison of the effect of ultrasound-guided thoracic paravertebral nerve block and intercostal nerve block for video-assisted thoracic surgery under spontaneous-ventilating anesthesia. Rev. Assoc. Med. Bras..

[17] Kadomatsu Y., Mori S., Ueno H., Uchiyama M., Wakai K. (2018). Comparison of the analgesic effects of modified continuous intercostal block and paravertebral block under surgeon’s direct vision after video-assisted thoracic surgery: A randomized clinical trial. Gen. Thorac. Cardiovasc. Surg..

[18] Patel K.M., van Helmond N., Kilzi G.M., Patel A., Bowen F.W., Shersher D.D., Trivedi K., Desai R.G. (2020). Liposomal Bupivacaine Versus Bupivacaine for Intercostal Nerve Blocks in Thoracic Surgery: A Retrospective Analysis. Pain Physician.

[19] McAnulty G., Cashman J., Keighley-Elstub C., Mellis M. (2010). Does intrathecal diamorphine improve pain relief after thoracic surgery?. J. Cardiothorac. Vasc. Anesth..

[20] Cohen E. (2013). Intrathecal Morphine: The Forgotten Child. J. Cardiothorac. Vasc. Anesth..

[21] Zakkar M., Tan C., Hunt I. (2013). 045 * The role of preoperative intrathecal diamorphine injection in thoracic surgery: Single-unit experience. Interact. Cardiovasc. Thorac. Surg..

[22] Liu N., Kuhlman G., Dalibon N., Moutafis M., Levron J.C., Fischler M. (2001). A randomized, double-blinded comparison of intrathecal morphine, sufentanil and their combination versus IV morphine patient-controlled analgesia for postthoracotomy pain. Anesth. Analg..

[23] Rawal N. (2023). Intrathecal opioids for the management of post-operative pain. Best Pract. Res. Clin. Anaesthesiol..

[24] Umari M., Falini S., Segat M., Zuliani M., Crisman M., Comuzzi L., Pagos F., Lovadina S., Lucangelo U. (2018). Anesthesia and fast-track in video-assisted thoracic surgery (VATS): From evidence to practice. J. Thorac. Dis..

[25] Granell-Gil M., Murcia-Anaya M., Sevilla S., Martínez-Plumed R., Biosca-Pérez E., Cózar-Bernal F., Garutti I., Gallart L., Ubierna-Ferreras B., Sukia-Zilbeti I. (2021). Clinical guide to perioperative management for videothoracoscopy lung resection (Section of Cardiac, Vascular and Thoracic Anesthesia, SEDAR; Spanish Society of Thoracic Surgery, SECT; Spanish Society of Physiotherapy). Rev. Esp. Anestesiol. Reanim..

[26] Piccioni F., Segat M., Falini S., Umari M., Putina O., Cavaliere L., Ragazzi R., Massullo D., Taurchini M., Del Naja C. (2018). Enhanced recovery pathways in thoracic surgery from Italian VATS Group: Perioperative analgesia protocols. J. Thorac. Dis..

[27] Mugabure Bujedo B. (2012). A clinical approach to neuraxial morphine for the treatment of postoperative pain. Pain Res. Treat..

[28] Bm B., Sg S., Au A. (2012). A review of epidural and intrathecal opioids used in the management of postoperative pain. J. Opioid Manag..

[29] Vijitpavan A., Kittikunakorn N., Komonhirun R. (2022). Comparison between intrathecal morphine and intravenous patient control analgesia for pain control after video-assisted thoracoscopic surgery: A pilot randomized controlled study. PLoS ONE.

[30] Bailey P.L., Rhondeau S., Schafer P.G., Lu J.K., Timmins B.S., Foster W., Pace N.L., Stanley T.H. (1993). Dose-response pharmacology of intrathecal morphine in human volunteers. Anesthesiology.

[31] Rathmell J.P., Lair T.R., Nauman B. (2005). The role of intrathecal drugs in the treatment of acute pain. Anesth. Analg..

[32] Pitre L., Garbee D., Tipton J., Schiavo J., Pitt A. (2018). Effects of preoperative intrathecal morphine on postoperative intravenous morphine dosage: A systematic review protocol. JBI Evid. Synth..

[33] Weibel S., Rücker G., Eberhart L.H., Pace N.L., Hartl H.M., Jordan O.L., Mayer D., Riemer M., Schaefer M.S., Raj D. (2020). Drugs for preventing postoperative nausea and vomiting in adults after general anaesthesia: A network meta-analysis. Cochrane Database Syst. Rev..

[34] Bendixen M., Jørgensen O.D., Kronborg C., Andersen C., Licht P.B. (2016). Postoperative pain and quality of life after lobectomy via video-assisted thoracoscopic surgery or anterolateral thoracotomy for early stage lung cancer: A randomised controlled trial. Lancet Oncol..

[35] Kampe S., Geismann B., Weinreich G., Stamatis G., Ebmeyer U., Gerbershagen H.J. (2017). The Influence of Type of Anesthesia, Perioperative Pain, and Preoperative Health Status on Chronic Pain Six Months after Thoracotomy—A Prospective Cohort Study. Pain Med..

[36] Bayman E.O., Parekh K.R., Keech J., Selte A., Brennan T.J. (2017). A Prospective Study of Chronic Pain after Thoracic Surgery. Anesthesiology.

[37] García-Tirado J., Rieger-Reyes C. (2012). Suture techniques of the intercostal space in thoracotomy and their relationship with post-thoracotomy pain: A systematic review. Arch. Bronconeumol..

